# High-Resolution Structure Analysis of Antibody V5 and U4 Conformational Epitopes on Human Papillomavirus 16

**DOI:** 10.3390/v9120374

**Published:** 2017-12-06

**Authors:** Jian Guan, Stephanie M. Bywaters, Sarah A. Brendle, Robert E. Ashley, Alexander M. Makhov, James F. Conway, Neil D. Christensen, Susan Hafenstein

**Affiliations:** 1 Department of Medicine, The Pennsylvania State University College of Medicine, 500 University Drive, Hershey, PA 17033, USA; fuyougj@gmail.com (J.G.); bobashley@gmail.com (R.E.A.); 2 Department of Pathology, The Pennsylvania State University College of Medicine, 500 University Drive, Hershey, PA 17033, USA; smb581@psu.edu (S.M.B.); ganzelly@gmail.com (S.A.B.); ndc1@psu.edu (N.D.C.); 3 Department of Structural Biology, University of Pittsburgh School of Medicine, 3501 5th Ave, Pittsburgh, PA 15260, USA; amm241@pitt.edu (A.M.M.); jxc100@pitt.edu (J.F.C.); 4 Department of Biochemistry and Molecular Biology, The Pennsylvania State University, Millennium Science Complex, University Park, State College, PA 16802, USA

**Keywords:** human papillomavirus, cryo-electron Microscopy (cryo-EM), conformational epitope, antibody neutralization, U4 and V5, competition, steric clash, complex, virus, fab

## Abstract

Cancers attributable to human papillomavirus (HPV) place a huge burden on the health of both men and women. The current commercial vaccines are genotype specific and provide little therapeutic benefit to patients with existing HPV infections. Identifying the conformational epitopes on the virus capsid supports the development of improved recombinant vaccines to maximize long-term protection against multiple types of HPV. Fragments of antibody (Fab) digested from the neutralizing monoclonal antibodies H16.V5 (V5) and H16.U4 (U4) were bound to HPV16 capsids and the structures of the two virus-Fab complexes were solved to near atomic resolution using cryo-electron microscopy. The structures reveal virus conformational changes, the Fab-binding mode to the capsid, the residues comprising the epitope and indicate a potential interaction of U4 with the minor structural protein, L2. Competition enzyme-linked immunosorbent assay (ELISA) showed V5 outcompetes U4 when added sequentially, demonstrating a steric interference even though the footprints do not overlap. Combined with our previously reported immunological and structural results, we propose that the virus may initiate host entry through an interaction between the icosahedral five-fold vertex of the capsid and receptors on the host cell. The highly detailed epitopes identified for the two antibodies provide a framework for continuing biochemical, genetic and biophysical studies.

## 1. Introduction

Human papillomavirus (HPV) is a non-enveloped DNA virus capable of causing anogenital warts and associated with cancers of the cervix, vagina, vulva, anus, penis, oral cavity and oropharynx (level II) [1,2,3]. Cancers attributed to HPV place a significant burden on the health of both men and women with cervical cancer presenting as the most common disease [4,5,6], although rates of oropharyngeal squamous cell carcinomas have risen disproportionately in men (7.6 per 100,000 persons compared to 1.7 in females) [7]. Commercial vaccines targeting the major capsid protein, L1, have been applied successfully to protect against high-risk HPV—for example, a new 9-valent vaccine for HPV was developed recently that protects against HPV types 6, 11, 16, 18, 31, 33, 45, 52 and 58 [3]. However, the efficacy of vaccines is still genotype specific and vaccines provide little therapeutic benefit against existing infection [8]. Understanding the antigenic nature of the HPV capsid offers an opportunity to discover structural features that are crucial to capsid integrity and conserved across species. Identifying neutralization-sensitive epitopes on the surface of the capsid adds to ongoing development of improved recombinant vaccines that maximize long-term antibody-mediated protection against multiple HPV Types [9,10,11,12].

Among more than 170 genotypes, HPV16 is the most prevalent high-risk type of HPV and has been a primary target for the development of prophylactic vaccines [13,14,15]. The infectious HPV16 has a T = 7 icosahedral capsid (55 to 60 nm in diameter) comprised of 72 L1 protein pentamers. The number of L2 minor structural proteins is uncertain but there may be up to 72 copies of L2 incorporated into the capsid [16,17,18,19]. Twelve L1 pentamers map to the icosahedral 5-fold axes (pentavalent capsomers), whereas the other 60 pentamers are located at the pseudo 6-fold axes (hexavalent capsomers). The apical surface of each capsomer is comprised of the antigenic loops (BC, DE, EF, FG and HI loops from each L1 protein) that connect eight antiparallel beta strands (BIDG and CHEF). This conformation forms the common jellyroll structural motif [20,21]. An extension of the L1 C-terminus, or C-terminal arm, links capsomers together and provides stability due to the formation of disulfide bonds between 175.CYS and 428.CYS from two neighboring L1 proteins [22,23].

Since the HPV life cycle depends on the differentiation of keratinocytes, the restricted tropism makes production and purification of high titer virus preparations for research problematic. Therefore, alternative HPV production methods have been developed for molecular biology and structural studies. Virus-like particles (VLPs) can be comprised of only the major structural protein (L1) or of the major and minor capsid proteins (L1/L2) but are not infectious since they are devoid of viral genome [24,25,26]. Pseudovirus (PsV) is comprised of both structural proteins, which are expressed along with a plasmid so that a copy of the plasmid DNA can be packaged as a mock genome [27]. Quasiviruses (QV) assemble a structurally complete capsid and are infectious since they package a copy of the cottontail rabbit papillomavirus genome (CRPV) [28]. All of these types of HPV capsids preserve the main attributes of the native capsid structure and have been used successfully for vaccine development and for studies of antigenicity, receptor usage, entry mechanisms and capsid structure. Quaisivirus has been used for the HPV16 work presented here and is referred to throughout as HPV.

First generated from mice in 1996, H16.V5 (V5) and H16.U4 (U4) are two distinctive monoclonal antibodies reactive to surface conformational epitopes on the intact HPV16 VLP [29]. V5 blocks the serological reactivity of human sera with the corresponding capsids and neutralizes authentic HPV16 in vitro [30,31]. V5 has become an especially important tool in inhibition-based HPV serological assays and is used extensively in major HPV vaccination trials. Mutational studies have suggested the FG and HI surface loops contribute to the V5 antibody binding sites [20,32,33,34]. Previous studies with U4 have shown this antibody to be type-specific, in that U4 neutralizes pseudotype HPV16 capsids but not authentic ones [20,31]. Further biological studies showed that U4 prevented virus binding to the cell surface but not to the extracellular matrix (ECM), suggesting a competition with cell receptor [33].

HPV16 capsid and Fab molecules have been used to form complexes for structural solution using 3-dimensional (3D) reconstruction techniques [11,35,36]. We previously solved cryo-EM reconstructions of HPV-V5 and HPV-U4 complexes with resolutions of 10–12 Å [11,12,36]. These modest resolution maps verified that V5 binds to L1 loops at the apical surface of the capsomer and U4 binds to the C-terminal arm of the L1 protein. The epitopes of the two Fabs were predicted from fitting of homology models. The previous work also provided evidence for neutralization mechanisms of super-stabilization and cross-linking for V5 and steric interference with cell attachment for U4.

Harnessing the revolutionary technology in cryo-EM image detection presented by direct electron detectors (DED) and reconstruction software programs, we recently investigated HPV and HPV-heparin complexes at near atomic resolution [37]. Besides identifying the heparin binding site around the five-fold vertex, this work specifically allowed us to define non-L1 densities in the HPV capsid, which might be interpreted as putative L2 protein densities. Due to the technological advance, atomic or near atomic resolution structures may correct or add to the information gained from previous low resolution structures and illustrate the importance of solving high resolution structures to be used to drive continuing research [38,39]. Here we present the near-atomic resolution structures of HPV-V5 and HPV-U4 complexes at 4.7 Å and 5.8 Å resolutions, respectively. With these high-resolution maps, the alpha carbon backbone of the Fab structure was built, the interaction between Fab and capsid was identified and Fab-induced capsid conformational changes were tracked for the first time. Specifically, the V5 stabilization mechanism was illustrated and the binding mode of the Fab identified without ambiguity, thus defining the epitope on an atomic level. Importantly, the epitopes identified here are based on structures built into the high-resolution density and the ensuing accuracy allows these footprints to supersede those identified previously by fitting Fab models into low resolution maps. Competition assays between V5 and U4 showed that binding of V5 prevents subsequent binding of U4, even though the V5 and U4 footprints do not overlap. We discuss mechanisms of conformational change and steric collision induced by V5 binding. The binding site of U4 was found to overlap with the recently determined heparin-binding site [37]. The preferential binding of U4 around pentavalent capsomers and not hexavalent capsomers suggests a model by which a capsid vertex may be selected during virus entry to interact with the host cell.

## 2. Materials and Methods

### 2.1. Preparation of Virus

HPV16 quasivirus containing L1 and L2 proteins and encapsidating a cottontail rabbit papillomavirus genome (CRPV) having the Simian vacuolating virus 40 (SV40) origin of replication were prepared as described previously [40,41,42]. Briefly, HPV16 sheLL plasmid (given by John Schiller, NIH) was transfected with linear CRPV/SV40ori DNA into 293TT cells. The preparation proceeded as described previously [43,44]. HPV16 matured overnight, pelleted by centrifugation, resuspended in 1 M NaCl 0.2 M Tris, pH 7.4. After CsCl gradient ultracentrifugation, the lower band was collected, concentrated and dialyzed against phosphate buffered saline (PBS), as described [11,12]. Concentrated HPV16 particles were applied to Formavar-coated copper grids, stained with 2% phosphotungstic acid (PTA) and observed by transmission electron microscopy (TEM) on a JEOL JEM 1400 electron microscope (Tokyo, Japan).

### 2.2. Preparation of Antibody and Fabs

Monoclonal antibody (MAb) production has been described previously [29,40,45]. MAb were purified from hybridoma supernatants using a protein A IgG purification kit (Pierce). Fab was digested and purified from the MAb using a Fab preparation kit (Pierce). Briefly, ~2 mg of each MAb was loaded over a Zeba desalting spin column (Pierce). Separately, V5 and U4 MAb were incubated with cysteine and papain at 37 °C for 3 h with end-over-end mixing. After this incubation, the sample was purified on a protein A column and BS was used to elute the Fabs. The purity of the Fab was assessed by absence of the Fc portion and the integrity of the Fab was determined by performing an ELISA (enzyme-linked immunosorbent assay). Antibody and Fab protein concentrations were estimated by taking optical density readings using absorbance spectrometry at a wavelength of 280 nm.

### 2.3. Cryo-Electron Microscopy

HPV16 was incubated with an excess of 4 Fab molecules for each of the predicted 360 binding sites per capsid for 1 h at room temperature and concentrated to 1.2 mg/mL in PBS buffer. A volume of 3 µL of virus-Fab complex was applied to Quantifoil holey carbon support grids (Quantifoil, Jena, Germany), blotted and plunged into liquid ethane using a Cryoplunge 3 (Gatan, Pleasanton, CA, USA). Low-dose micrographs were recorded using an FEI Polara G2 microscope operating at 300 kV. Images were collected under the control of the E Pluribus Unum software program (EPU) using an FEI Falcon 2 direct electron detector with 1.4× post-column magnification and a nominal magnifications of 59,000× for HPV-V5 and 93,000× for HPV-U4, yielding calibrated pixel sizes at the sample of 1.75 Å and 1.15 Å, respectively. Defocus ranges of 0.65–3.56 µm and 0.26–5.18 µm (Table 1) were measured for micrographs of HPV-V5 and HPV-U4, respectively. REgularized LIkelihood OptimizatioN (RELION), AUTO3DEM and Electron Micrograph ANaly-sis (EMAN2) program software suites were used for image processing and 3D reconstructions [46,47,48].

### 2.4. Image Processing

Whole frame alignment was carried out using MotionCorr to account for stage drift [49]. The microscope contrast transfer function parameters were estimated for each micrograph using ctffind4 [50]. Semiautomatic particle selection was performed using EMAN2 e2boxer.py to obtain the particle coordinates, followed by particle extraction, linearization, normalization, apodization and 3-D reconstruction of the images using auto3DEM 4.05 following the gold standard method of initiating the process by splitting the data set into two independently-refined halves [46,51]. The sharpening of the high resolution information was performed using EMBFACTOR [52,53]. Reported resolutions were estimated according to the gold-standard Fourier shell correlation (FSC) = 0.143 criterion [54].

### 2.5. Model Building

The atomic model of the HPV16 L1 capsomer with the C-terminal arm truncated (PDB ID, 3OAE; residues 20–403, 438–474) was fitted as a rigid body and propagated to fill an asymmetric unit of the HPV16 capsid (PDB ID, 3J6R; residues 9–486) [55,56]. This asymmetric unit was used as a guideline during model building [57], which proceeded de novo to place the missing C-terminal arm residues 404–437 and the N- and C-termini residues using the software program Coot [58]. Building of the Fab structure was initiated by fitting a previously published variable domain model (V5 PDB ID 3J7E; U4 PDB ID 3J6R) and the constant domain of 3GK8 using Coot [12,35,58]. Icosahedral symmetry was applied to the refined asymmetric unit model including Fabs to generate the capsid-Fab complex through Situs [59,60]. The capsid atomic model was then further refined iteratively using Phenix Real Space Refinement program [61]. At each iteration, the best model was visually inspected in Coot and adjusted manually accordingly to the best fit into the density and guided by torsion restraints, planar peptide restraints and Ramachandran restraints. The quality of the final model was evaluated by Molprobity (http://molprobity.biochem.duke.edu/) [62] (Table 2 and Table 3). Contacts between the fitted Fab and L1 protein structures were identified using UCSF Chimera with the criteria for van der Waals (VDW) overlap distances set at −0.4 and 0.0 Å. Clashes between atoms were defined by any overlap of 0.6 Å or more [56].

### 2.6. Magnitude Ratio Calculation

Our native HPV16 map (EMDB-6620) was subtracted from the HPV-V5 and HPV-U4 complex maps separately to identify difference density corresponding to V5 Fab and U4 Fab, respectively. The difference maps were used as a guide to mask the Fab densities from HPV-Fab complex maps for comparison of their average density with that of the capsid (density/pixel for the whole box size) using the mask function of UCSF Chimera. The average density was further divided by the volume of capsid and Fab densities, respectively, to obtain a final density magnitude value. To compare the hexavalent V5 Fab and pentavalent V5 Fab, their respective densities were isolated using the segmentation function of UCSF Chimera followed by the method described above.

### 2.7. Competition Capture ELISAs

Purified mAbs V5 or U4 were bound to the wells of a 96-well microtiter plate in 50 µL 50 mM Sodium Carbonate pH 9.6 and incubated overnight at 4 °C. The wells were washed with wash buffer (PBS supplemented with 0.05% Tween-20) following the overnight incubation and in between all subsequent additions to the wells. Blocking buffer (5% non-fat milk) was added to the wells for one hour and during the one hour block, HPV (quasivirus) (500 ng/well) and serial dilutions of mAb were incubated together in blocking buffer. Following the removal of the blocking buffer, HPV-mAb complex was added to the wells and incubated for 30 min. We could not assess capture of the complex directly by probing for the complexed mAb because it was titrated and low concentrations of the complex mAb would inevitably result in low levels of detection. Capture of the HPV-U4 complex was therefore determined indirectly through the binding of a V5 IgA class/isotype switch-variant (10 µg/mL) which differs phenotypically from the original mAb clone due to rearrangements in the heavy chain genes and an alkaline-phosphatase (AP) conjugated anti-IgA secondary antibody (Southern Biotech). AP signal was developed with p-Nitrophenyl Phosphate (Sigma, St. Louis, MO, USA). Due to the fact that the V5 IgA mAb shares the same epitope as the original V5 clone (IgG2b), we could not directly use the V5 IgA mAb as a probe for detection of captured HPV-V5. Instead, we assessed HPV capture by probing for both V5 isotypes.

Cryo-EM maps for the HPV-V5 and HPV-U4 complexes are deposited in the EM database (www.emdatabank.org/) with accession numbers EMD-8243 and EMD-7136, respectively. Coordinates for each atomic model of the asymmetric unit of the two complexes with V5 and U4 Fab molecules were deposited in PDB 6BT3 and PDB 6BSP, respectively.

## 3. Results

### 3.1. Cryo-EM Reconstructions of HPV-V5 Fab and HPV-U4 Fab Complexes

**Figure 1:**
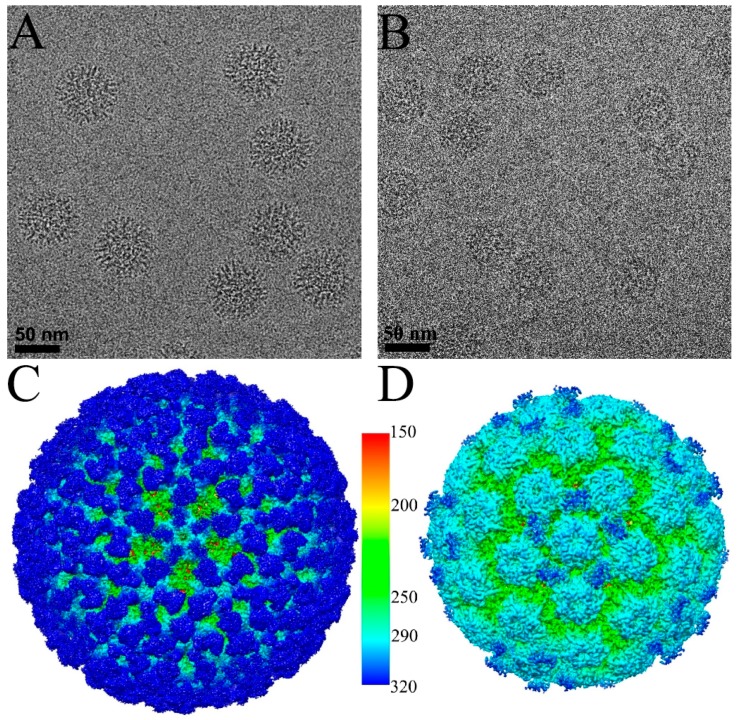

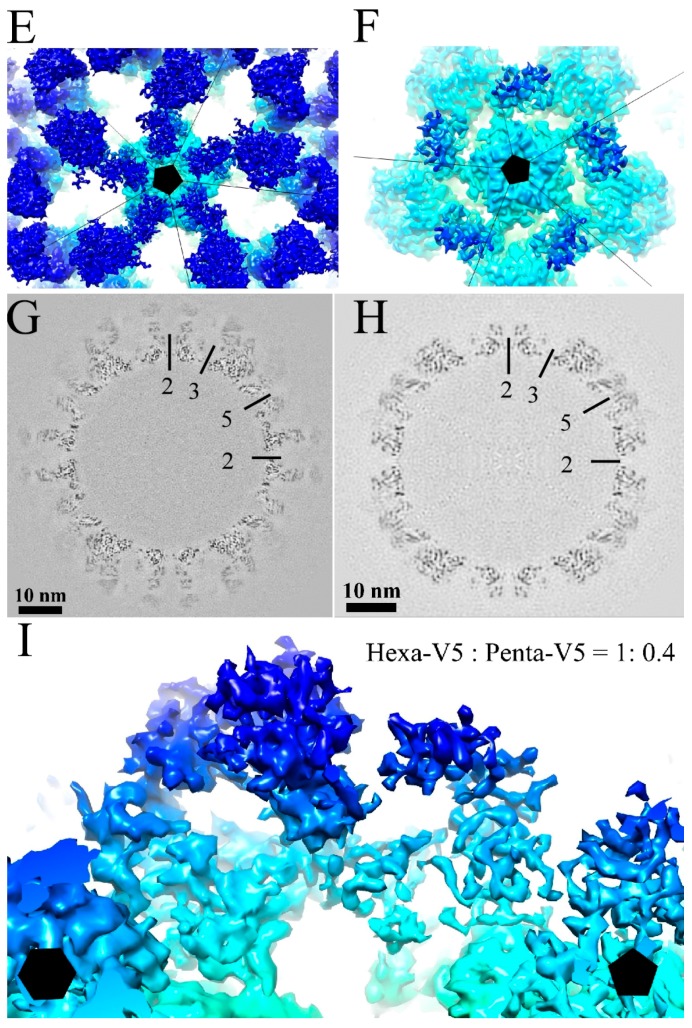
Cryo-electron microscopy (Cryo-EM) reconstructions of HPV16-V5 and HPV16-U4. Representative regions of cryo-EM micrographs of the HPV16-V5 (**A**) and HPV16-U4 (**B**) virus-fab complexes; (**C**,**D**) the surface rendered 3D maps of HPV16-V5 (**C**) and HPV16-U4 (**D**) were radially colored according to the distance from the center (color key in Ångstroms) of the capsid and surface rendered at 1σ; (**E**,**F**) the zoom-in view of the five-fold vertex (black pentagon) of V5 (**E**) and U4 (**F**) maps shows the density corresponding to bound Fab molecules (dark blue); (**G**,**H**) the central sections through the cryo-EM density maps show the quality of the reconstructions. Capsids were cut vertically through the two-, three- and five-fold icosahedral symmetry axes (black lines), with the central two-fold axis appearing at the 12 o’clock position; (**I**) the V5 Fab densities associated with the hexavalent (black hexagon) and pentavalent (black pentagon) capsomers showed different magnitudes. The magnitude ratio between hexavalent Fab (Hexa-V5) and pentavalent Fab (Penta-V5) was measured as 1:0.4.

The purified V5 Fab molecules formed stable complexes with HPV16 virions. Cryo-EM images of frozen hydrated complexes revealed a seemingly homogenous population of ~700 Å diameter spheres labeled uniformly with V5 Fab branch-like densities (Figure 1A). No empty capsids were observed, although some unbound V5 Fab was apparent in the background due to the incubation of Fab in excess of binding sites. For the HPV-U4 complexes, binding with an excess of U4 Fab molecules did not change the capsid appearance significantly but resulted in a slightly blurred capsid circumference (Figure 1B). The size of HPV-U4 complex appeared about the same as the HPV16 unbound particles with a diameter of ~580, suggesting no obvious gross conformational changes and indicating the U4 is bound into the canyon region.

**Figure 2:**
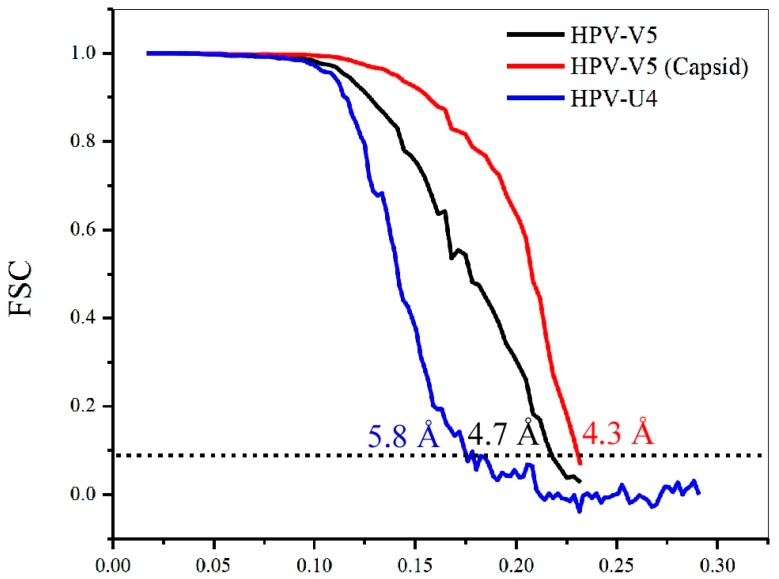
Plot showing the Fourier shell correlation (FSC) versus spatial frequency of the icosahedrally-averaged reconstructions for HPV16-V5 complex (with fab included), HPV16-V5 capsid (with fab masked out) and HPV16-U4 complex. The resolution of the reconstructions was assessed where the FSC curve crossed a correlation value of 0.143; the x axis is 1/Å.

Using the gold standard method of image reconstruction, the structures of HPV-V5 and HPV-U4 complexes were determined to resolutions of ~4.7 Å and ~5.8 Å, respectively, where the Fourier shell correlation (FSC) dropped below 0.143 (Figure 2). However, when the V5 Fab densities for V5 were excluded from the calculations, the local resolution of capsid reached ~4.3 Å (Figure 2), which was comparable to our recent HPV16 reconstruction [37].

The bound V5 Fab moieties could be recognized easily as spikes on the surface of the HPV cryo-EM density map and occupied nearly all binding sites on the capsid (Figure 1C,E). However, there was an apparent density difference between Fabs occupying hexavalent capsomer and pentavalent capsomers, as has been noted previously [35,36] (Figure 1E,I). The pentavalent Fab density was not continuous and the magnitude was significantly weaker (60%) than the hexavalent Fab density.

In the HPV-U4 complex map, density regions corresponding to the U4 Fab were observed around the pentavalent capsomers at each five-fold vertex of the capsid (Figure 1D) as has been seen previously at lower resolution [12]. Fab density was located in each of the five canyons formed between pairs of hexavalent capsomers and the pentavalent capsomer, resulting in a total of 60 Fab binding sites per capsid. However, as the U4 Fab density was 70% of the HPV16 capsid, we estimate that on average only 42 of the 60 sites per capsid were occupied by U4 Fabs. Central sections through the density maps illustrated the high quality of the reconstructions and identified the location of Fab densities according to the 2-, 3- and 5-fold symmetry (Figure 1G,H).

### 3.2. Building the V5 Fab and L1 Protein Structures into the High-Resolution Cryo-EM Maps

**Figure 3:**
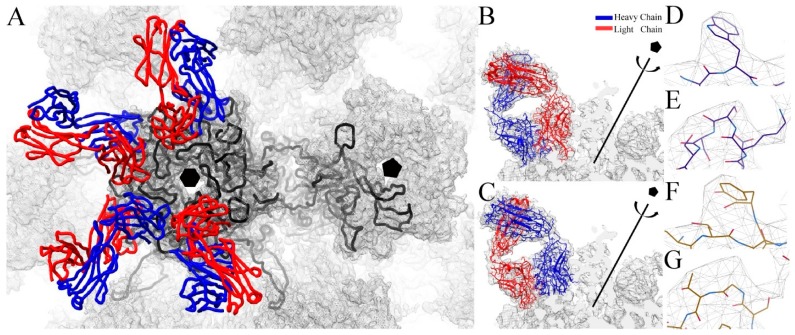
V5 Fab and L1 protein structures. (**A**) The asymmetric unit used for refinement includes six L1 proteins (black wire), five L1 from the hexavalent (black hexagon) and one L1 from the pentavalent (black pentagon) capsomers and four V5 fabs with heavy chain (blue) and light chain (red) shown in wire. V5 Fab was built in both possible orientations; (**B**) with heavy chain (blue ribbon) facing outward from the center of the capsomer and (**C**) with the light (red ribbon) chain facing outwards. Close up views show the fit of specific residues that aided in the identification of the correct binding mode; (**D**) the side chain of 59H.TRP fitted well into the density of the heavy chain outwards build; however light chain outward placed 59L.TYR into the same density and a poor fit; (**F**) similar results were also observed for H74-H77 residue sequence in heavy chain facing out mode (**E**) compared to the light chain facing out mode (**G**) of unfilled density.

The predicted web based antibody model (WAM) structure model of the Fab variable domain was combined with the constant domain of a published homologous crystal structure (PDB ID 3GK8) [63] and fit into the corresponding cryo-EM density. The Fab model was built by stepping through the fitted structure and correcting all sections and discernable side chains that were out of density. The L1 proteins inside the hexavalent and pentavalent capsomer were built independently using the crystal structure (PDB ID 3OAE) as a guide and correcting and adding residues that could be uniquely assigned to density. Due to steric limitations, only four V5 Fabs were included in the asymmetric unit, which was comprised of five hexavalent L1 proteins and one pentavalent L1 protein (Figure 3A). Refinement of the asymmetric unit was followed by global refinement in the context of the entire capsid. Verification of the resulting structure was evaluated with Molprobity (Table 2) (Methods).

Due to the pseudo-two-fold symmetry of the Fab molecule, the V5 Fab could be fitted with the heavy chain facing outwards away from the capsomer center or with the light chain facing outwards (Figure 3A,B). The V5 molecule structure was built separately in both possible binding modes. Previously, based on a poorer resolution map, the proposed model for V5 binding was in the heavy chain-outwards mode of binding [11]. This mode was confirmed here using the new near-atomic model of V5 Fab (Figure 3D–G). Examples that verify the orientation of Fab include the side chain of 59H.TRP, which fit better in the heavy chain outwards structure, since the light chain outward mode would place a 59L.TYR poorly into the same density (Figure 3D,F). A similar result was also observed in the better fit of H74-H77 compared to a poorer fit of L74-H77 in the same density (Figure 3E,G).

**Figure 4:**
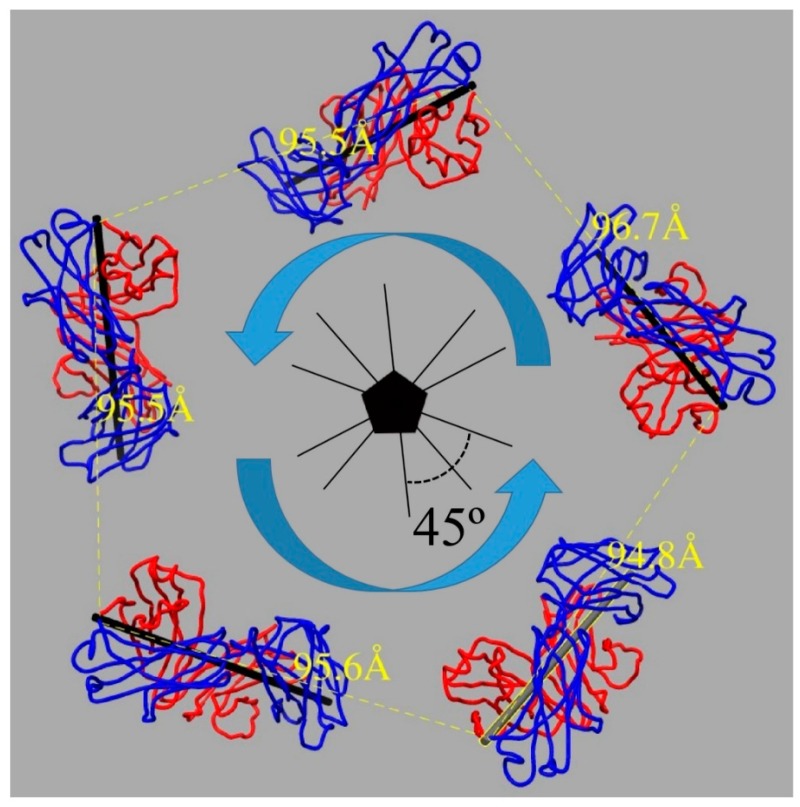
U4 Fab bound the HPV16 capsid in a heavy chain (blue) followed by the light chain (red) mode around each five-fold vertex in a counterclockwise direction (blue arrow). The distance between the neighboring heavy chain C-termini (yellow) at the constant domain was measured to discriminate between monovalent and bivalent binding of U4 Mab. The pseudo-two-fold axis of the Fab (black stick) is indicated and the angle between neighboring axes was 45 degrees.

The structure of U4 Fab was produced using the same method as for V5. The L1 asymmetric unit structure produced from the HPV-V5 complex was used to initiate building L1 in the HPV-U4 complex. Since U4 Fab only bound in the canyon around the pentavalent capsomers, the asymmetric unit for HPV-U4 included six L1 proteins and one U4 Fab. This unit was used to generate the whole capsid-U4 structure for Phenix global refinement. U4 Fab was built with the heavy chain relative to the light chain (heavy chain followed by the light chain) around the five-fold vertex in a counterclockwise direction (Figure 4) [12]. The Molprobity score was also used to evaluate U4 model building (Table 3).

### 3.3. V5 Binding Induces Order to Capsomer Loops and Improves Resolution Significantly

**Figure 5:**
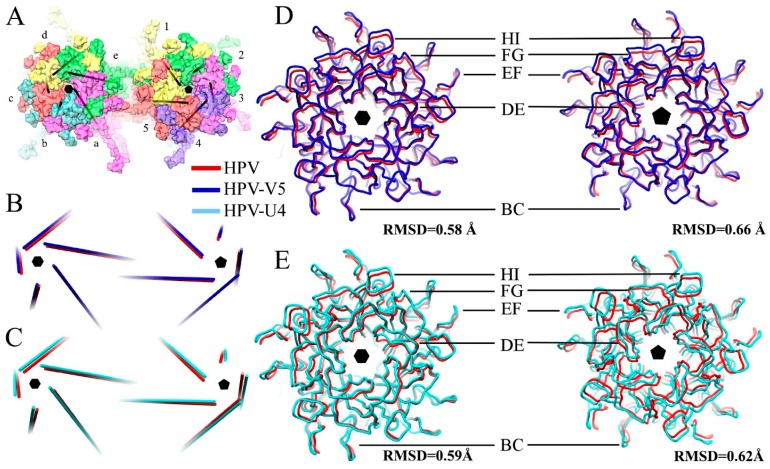
Fab binding induces conformational changes to the HPV16 capsid. (**A**) The L1 proteins in the pentavalent (labeled 1–5) and hexavalent (labeled a–e) capsomers are colored coded individually as pink, cyan red, yellow and green with the mass axis (black) depicted for each L1; (**B**,**C**) the mass axis arrangement in HPV (PDB ID: 5KEP) (red) is superimposed with those of HPV-V5 (Blue) and HPV-U4 (green) (**D**) (blue) and those from the HPV-U4 (**E**) (cyan) maps, which illustrates the movement of surface loops on the capsomers (37).

**Figure 6:**
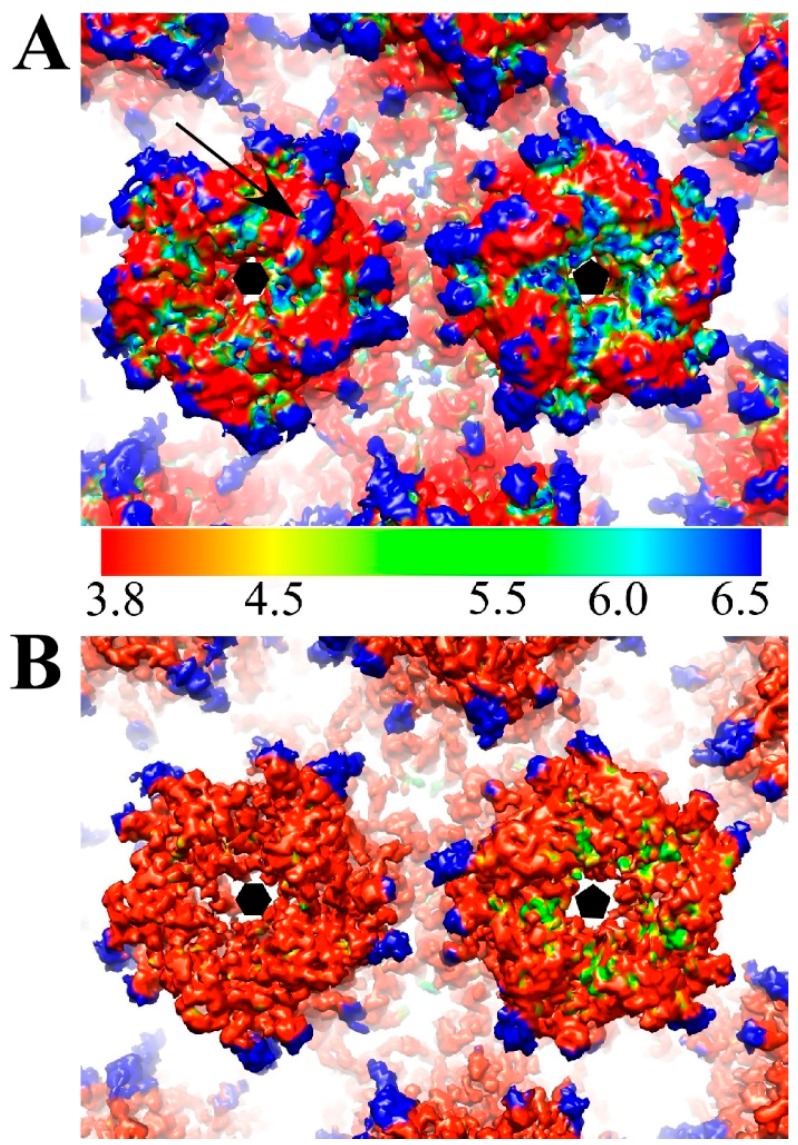
Surface rendered maps of HPV (EMDB-6620) (**A**) and HPV-V5 (**B**) are colored according to local resolution (color key 3.8–6.5 Å resolution) and zoomed to show hexavalent (black hexagon) and pentavalent (pentagon) capsomers (37). Notably the region of the FG and HI loops (black arrow) is quite flexible prior to Fab binding as attested by the low resolution. After Fab binding the entire apical capsomer surface has been stabilized and has a local resolution of ~3.8 Å.

The mass axis of L1 proteins in pentavalent and hexavalent capsomers in the HPV and the HPV-Fab complex maps were determined (Figure 5A,B) to track potential rotational and translational movements of L1 proteins after Fab binding (Table 4). Binding with V5 induced the L1 protein to rotate only slightly outwards (0.3 degrees) from the center of the capsomer; however, L1 moved as a rigid body away from the capsomer center 2.5 Å. The arrangement of surface loops remained nearly the same and only slight translational movements as a rigid body were measured (Figure 5D). Superimposition of the pentavalent and hexavalent capsomer structures from the HPV-V5 and HPV16 reconstructions were essentially the same with statistically negligible root-mean-square deviation of atomic positions (RMSD) (0.58 Å and 0.66 Å, respectively for hexavalent and pentavalent capsomers). After masking out the Fab densities from the map, the capsid shell resolution was nearly the same (4.3 Å) (Figure 2) as our previously solved HPV16 map [37]. However, the local resolution of the pentavalent and hexavalent capsomers was significantly improved, which indicates structural ordering of the loops was induced by binding with Fab molecules. Especially for the apical capsomer surface, compared to HPV virions, both pentavalent and hexavalent complex capsomers had nearly 2 to 3 Å better resolution after Fab binding (Figure 6). Compared to the native HPV16 map, there are some regions of lower resolution in the complex (Figure 6A, blue arrow) where FG and HI loops are located. The only capsid location that showed no change after Fab binding was at the rim of the capsomer, at the location of the EF and BC loops, where the local resolution stayed the same after binding with Fab.

### 3.4. U4 Fab Binding Suggests MAbs Bind Monovalently

The newly built models of U4 Fab and the L1 protein were used to evaluate the binding mode for the entire bipartite antibody molecule. For an antibody to be able to bind bivalently, the distance between the C-terminal Cα atoms of adjacent heavy chains is typically in the range of 25–29 Å [64,65,66]. The angle between two Fab arms in a typical IgG antibody can reach 120° [67,68]. The distance between Fabs in the HPV-U4 complex was 96 Å between two neighboring U4 Fabs bound around the pentavalent capsomer, whereas the angle between two neighboring Fabs was 45°. These distances and angles are consistent with monovalent binding for MAb U4 (Figure 4), which suggests the Fc portion of an intact U4 antibody would allow the variable regions to bind two different capsids, thereby crosslinking capsids as a potential neutralization mechanism.

### 3.5. Assessing Capsid Conformational Changes Initiated by U4 Binding

**Figure 7:**
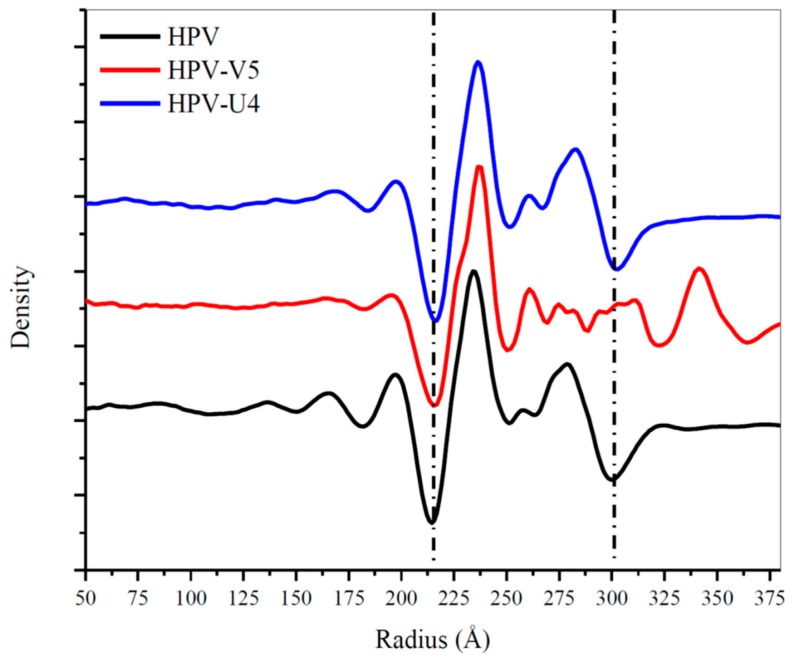
Radial density distributions from center of the map to the edge of the capsid for HPV16 (EMDB-6620) (black), HPV16-V5 (red) and HPV16-U4 (blue) maps. The two dotted lines designate the capsid range.

The U4 Fab was observed only in the canyon between hexavalent and pentavalent capsomers with most of its body inside of the inter-capsomeric space (Figure 1F). The radial density plots of the density maps indicated no significant difference in diameter between HPV16 and the HPV-U4 complex map (Figure 7) suggesting that U4 Fab binding did not trigger a change in the overall size of the capsid. The mass axis of L1 proteins in pentavalent and hexavalent capsomers in the HPV and the HPV-U4 Fab complex maps indicated negligible rotational and translational movements of L1 protein as a rigid body after U4 binding. The measurement of L1 protein rotation away from the capsomer center was ~1°, with a 2 Å movement from the center (Table 4). The measurements for L1 protein differences in hexavalent and pentavalent capsomers were approximately the same. The RMSD value of L1 proteins in hexavalent and pentavalent capsomers were less than 1 Å when superimposed with the capsomer structures of native HPV16 (EMDB-6620). Thus, at this resolution the measurements are below what can reasonably be considered actual movement induced by Fab binding.

### 3.6. V5 and U4 Bind to Different Sites

**Figure 8:**
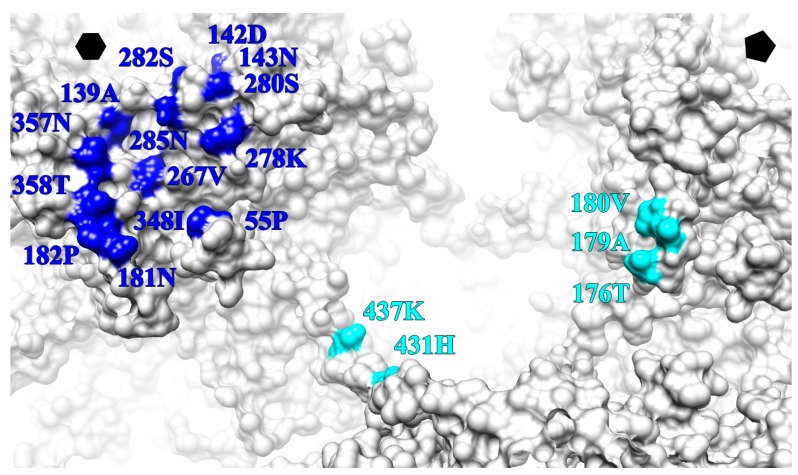
Zoomed view of a surface rendered HPV16 capsid (grey) map illustrates the epitope of V5 (blue) and U4 (cyan) on the surface. The V5 epitope is shown mapped to the hexavalent (hexagon) capsomer, whereas the U4 epitope lies between the hexavalent and pentavalent (pentagon) capsomers.

Atomic models of L1, V5 and U4 fitted into the cryo-EM density were used to define the antibody binding sites (Table 5). The epitope of V5 was distributed among BC, DE, EF, FG and HI loops contributed by two neighboring L1 proteins within one capsomer. As the major contributor to residues in the binding site, the FG loop provided five residues (267.VAL, 278.LYS, 280.SER, 285.ASN), whereas three residues mapped to the HI (348.ILE, 357.ASN, 358.THR) and DE (139.ALA, 142.ASP, 143.ASN) loops. All of the binding sites were exposed and located at the topmost apical surface of the capsomer (Figure 8). The binding site for the U4 Fab was located deep in the canyon and significantly different than reported previously [12]. Specifically the U4 epitope was comprised of residues from the C-terminal arm of a hexavalent L1 protein (431.HIS and 437.LYS) located adjacent to a pentavalent capsomer and three residues from the EF loop (180.VAL, 179ALA, 176THR) of the pentavalent L1 at the edge of the capsomer crown (Figure 8). The V5 and U4 antibody binding sites did not interfere or overlap with each other. Since a total of 14 residues participated in the binding with V5 compared with five residues for U4, the interaction between HPV16 capsid and V5 was predicted to be stronger than the interaction with U4.

### 3.7. V5/U4 mAb Competition Is Sequentially Dependent

**Figure 9:**
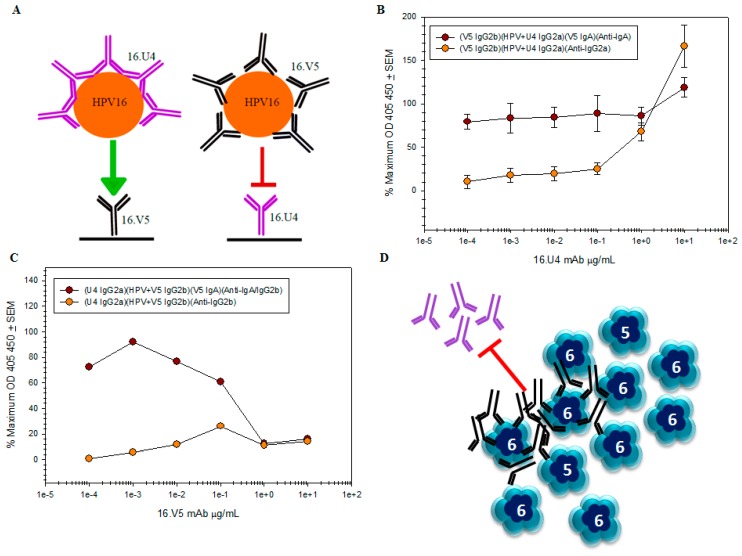
U4 and V5 bind different L1 epitopes but likely steric hindrance prevents U4 capture in ELISA. Two different HPV preparations were assayed and the mean of three independent assays, each with duplicates, are shown. Captured HPV-mAb complex is graphed as a percentage of maximum HPV capture in the absence of mAb. Parentheses ( ) in the figure legends denote the sequential additions of mAb or HPV-mAb complex and the mAb isotype (**A**) Schematic of 16.V5 and 16.U4 competition capture ELISAs. The capture antibody was bound to the well of a microtiter plate and complexed HPV-mAb was added in attempt to bind and immobilize the complex; (**B**) high concentrations of U4 did not impede capture of the HPV16-U4 complex by the V5 mAb. The percentage of captured HPV detected by isotype switch-variant V5 IgA 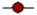 remains the same regardless of antibody concentration, while it is apparent that the amount of U4 detected by the anti-IgG2a secondary mAb titrates 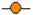; (**C**) capture of the HPV-V5 complex by the U4 mAb 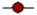 was ablated at high concentrations of V5. The mAb interference and the inability to capture HPV is also evident by the failure to directly detect V5 mAb 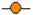; (**D**) schematic of pentavalent and hexavalent L1 pentamers (blue) demonstrating steric hindrance of V5 mAb (black) with the U4 mAb (purple). Preliminary binding of the V5 mab to the vertices of hexavalent capsomers creates an “umbrella” over the U4 epitope and thus prevents the secondary binding of U4 mAb.

Although the V5 and U4 footprints do not overlap, V5 binding induces conformational changes in the form of ordering flexible loops comprising the capsomers. To assess further the V5 and U4 epitopes biochemically and the consequences of binding, we performed a competition assay with the V5 and U4 mAbs. Competition was assessed by a capture ELISA in which U4 was used to capture HPV-V5 complex and V5 was used to capture HPV-U4 complex (Figure 9A). HPV-U4 was successfully captured by V5 mAb, confirming the non-overlapping nature of the two epitopes (Figure 9B). When the U4 mAb was used to capture the HPV-V5 complex, the amount of captured complex was diminished at the highest concentrations of V5 mAb (Figure 9C). The detection of captured HPV was only recovered at lowest dilutions of V5 mAb to 0.1 µg/mL.

## 4. Discussion

### 4.1. The Near-Atomic Resolution Map Allowed Building of the 50 kDa Fab Structure

The high resolution achieved for the complex maps allowed us to build the structures of the L1 protein, including the C-terminal arm and the relatively small 50 kDa V5 Fab molecule. Here we have overcome the difficulty of reconstructing a small molecule by incubating virus particles together with the small Fab molecules and solving the structure of the icosahedrally symmetric complex at high resolution. The structure of Fab was then built into the corresponding density. Thus, the large virus acted as a symmetric scaffold presenting the smaller Fab molecules for structural solution. This approach suggests a general use for symmetric virus capsids serving as a platform for solving the structure of small bound proteins and allows us to solve Fab structures without crystallography.

### 4.2. V5 Neutralizes by Stabilization

Our previous results suggested a super-stabilization neutralization mechanism for V5 based on the interpretations developed from a WAM model of V5 and a 10 Å resolution complex map [35]. The new higher resolution map presented here allowed building of both V5 and HPV16 L1 protein. This improvement allowed us to measure potential movements within the capsid that might have been induced by V5 binding. The small magnitude of the measurements could not be interpreted as movement of the L1 protein since they were well below the level of detection at this resolution (Figure 5). However, stabilization induced by Fab binding was observed readily as the significantly improved resolution of the capsid (Figure 6), which indicated less flexibility of the surface loops was possible after an interaction with V5. The stabilization of capsid likely inhibits conformational changes that are known to be essential for virus host entry and infection (3–9) [69,70].

### 4.3. V5 Preferentially Interacts with the Hexavalent Capsomer

Due to the steric hindrance between V5 binding sites on adjacent hexavalent and pentavalent capsomers, only one V5 Fab can bind in either position [11,35]. However, occupation of the hexavalent versus pentavalent site is not split evenly. Upon measuring the density magnitude ratio (pentavalent Fab/hexavalent Fab = 40%) (Figure 1I), V5 was found to bind preferentially to the hexavalent L1 protein binding site. The quasi-equivalence of the T = 7 icosahedral capsid and previous studies have predicted structural differences between the L1 molecules occupying hexavalent and pentavalent capsomers [35,36]. The residues of the V5 epitope were found here to be distributed differently in the pentavalent capsomer than the hexavalent capsomer due to the different L1 environments. Thus, the preferential binding of V5 for the hexavalent capsomers effectively leaves the pentavalent capsomers free to interact with host proteins. Previous biological studies showed that V5 did not prevent attachment of capsids to the cell surface but inhibited the internalization of cell surface-bound capsids [33]. Thus, the binding pattern of V5 suggests that HPV16 likely binds to the cell surface via pentavalent capsomer interactions to initiate an infection.

### 4.4. U4 Blocks the Accessibility of Pentavalent Capsomer Sites

Based on previous biological studies, binding with U4 directly interferes with the virus attachment to the cell surface, as shown by the inability of U4 to neutralize the virus post-attachment [33]. This phenomenon suggested the HPV16 capsid attaches to the cell surface receptor near the U4 binding site in the canyon around pentavalent capsomers. This model is consistent with our recent work that identified the site of heparin binding [37]. As a primary cell receptor for HPV16, the heparin molecules were located around the pentavalent capsomer near the canyon in between the neighboring EF and BC loops. Two of the five residues in the epitope of U4 overlapped with the known heparin binding site. Thus, even though the geometry of binding might allow intact U4 antibody to crosslink capsids, we propose that direct interference with receptor binding and blocking the accessibility of the canyon are the main neutralization mechanisms for U4.

### 4.5. The Conformational Epitopes of V5 and U4 Were Identified Using the High-Resolution Maps

Through previous immunological and structural studies, the epitopes of V5 and U4 were predicted as multiple loops with participation from different copies of the L1 protein; however, these previous epitopes were predicted based on fitting of homology models into the more globular envelope of poorer resolution maps. Here we identified the epitopes of both V5 and U4 from the building directly into near-atomic resolution maps. Thus, here we present the new, accurate epitopes of V5 and U4, which supplant the predicted ones presented previously. Fewer residues were found in the epitope of V5 than previously predicted as only nine residues were assigned to the new epitope. However, the epitope of U4 was significantly different from that previously predicted from a poorer resolution map. The C-terminal arm from one hexavalent protein and the EF loop from a pentavalent L1 protein contributed to the binding with U4, which bridged the two neighboring proteins.

**Figure 10:**
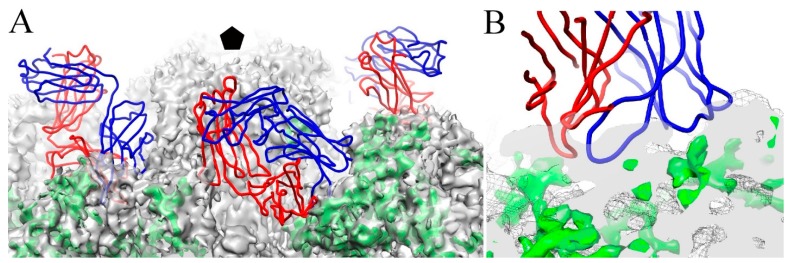
U4 may interact with the minor structural protein, L2. (**A**) The U4 Fab structure (heavy and light chain, blue and red, respectively) was superimposed onto putative L2 density identified previously (green) and the HPV16 capsid (grey mesh) (EMDB-6620) (37). The pentavalent capsomer is indicated in black pentagon. The zoomed view (**B**) shows the interface of the U4 variable domain with putative L2 density (green).

In our recent work with the 4.3 Å resolution map of HPV16, the putative L2 protein density was found to occupy the outer side wall of the capsomer, with some minor portions at both surface and the base. Even though U4 was raised against L1-only capsids, the U4 footprint overlaps the position of L2 density (Figure 10), suggesting that regions of L2 protein are buried when U4 is bound to the capsid. This possibility may provide another mechanism for U4 neutralization by stabilization or sterically occluding regions of L2 protein necessary for entry. Thus, bound U4 might inhibit the exposure of the L2 N-terminus for furin cleavage or other essential L2 related events required during endocytosis [5,6,10,11,12,13].

### 4.6. The V5/U4 Conformational Epitopes Are Non-Overlapping but V5 Outcompetes U4

Competition capture ELISAs confirmed that V5 and U4 bind two distinct regions of the viral capsid as V5 captured 100% of HPV particles complexed with U4. Although these antibodies bind disparate epitopes, the interference observed when HPV-V5 complexes were tested for capture by U4 mAb suggests a steric collision of intact antibody. The possible steric hindrance by the V5 Fc may be due to the binding angle of the V5 Fab at 47° Saturating concentrations of V5 could therefore create an “umbrella” over the capsid canyons, rendering the U4 epitope impenetrable to U4 mAb and subsequent capture of the HPV-V5 complex impossible (Figure 10D). The surface-saturating effects of V5 leads us to conclude that U4 binding in the canyons of pentavalent capsomers is hindered by the steric blockade of the V5 Fc when sufficient saturation of the capsid has taken place. The opposite effect is not possible because U4 binds deep into the canyon and cannot occlude the more apically positioned V5 binding site.

When HPV was incubated with low ratios of V5, U4 was also able to bind to the capsid, indicating that binding of V5 is not inducing conformational changes that affect subsequent U4 binding. However, we cannot rule out that higher V5:virus ratios might lead to global capsid changes that do alter the U4 binding site. We have observed in our previous V5 map and the near atomic resolution map presented in this paper that V5 binding induces hyper-stabilization of the capsid. V5 Fab binding was shown to result in more ordered density of the L1 C-terminal arm, suggesting that V5 functionally inhibits capsid flexibility and stabilizes the C-terminus [35], which is the site of U4 binding. V5-induced stabilization of the C-terminal arm therefore might have the potential to modify the U4 epitope but only when most or all of the V5 binding sites are occupied. The extent of capsid stabilization positively correlates with the number of bound V5 Fabs suggesting that if V5 inhibits U4 binding through conformational changes it may only be able to do so under saturating conditions of V5.

The data presented herein elucidate function of two critically important MAbs, particularly V5, which have been used extensively in HPV vaccine development, quality control assays to define VLP vaccine integrity during storage and preservation and immunogenicity. By taking advantage of the technological advances in cryo-EM, we have solved the structures of HPV16-Fab complexes at near-atomic resolution and thus for the first time, have been able to unambiguously define the epitopes of V5 and U4. To clarify, the V5 and U4 antibody footprints presented in this work supersede any of our previously reported epitopes based on fitting models into EM maps. Although this method has been cutting edge, the cryo-EM resolution revolution has ushered in a new era offering structural biologists new accuracy. This accuracy has afforded a more thorough investigation of the Fab-induced stabilization mechanisms and has allowed us to learn more about mechanisms of neutralization in addition to viral entry mechanisms. Specifically the V5 preferential binding of hexavalent capsomers leaves the five folds vertices (pentavalent capsomers) open even during V5 saturating conditions, which may be directly linked to the fact that V5 does not inhibit host cell attachment. Given that U4 binds exclusively to the pentavalent capsomers, the structure and function of the two antibodies is consistent with an HPV entry model whereby the virus gains entry to the cell via initial interactions mediated by residues that map to the pentavalent capsomers.

## Figures and Tables

**Table 1:** Cryo-EM image reconstruction data.

	HPV-V5	HPV-U4
Number of Micrographs	1899	8899
Defocus level range (µm)	0.65–3.56	0.26–5.18
Number of Particles Selected from Micrograph	27,095	58,571
Number of Particles Selected for reconstruction	17,612	35,151
Final Resolution	4.7	5.8

**Table 2:** Molprobity Statistics for HPV-V5.

Asymmetric Unit	Six Subunits (L1 Protein)	Cryo-Electron Microscopy (Cryo-EM) Model 3732 Residues at 4.7 Å Resolution after Phenix Global Refinement
Density agreement	Correlation Coefficient	0.85
All-atom contacts	Clash score * (all atoms)	31.04 (14th percentile)
Protein geometry	Poor rotamers	0.13%
	Favored rotamers	99.25%
	Ramachandran outliers	1.05%
	Ramachandran favored	90.52%
	Molprobity Score	2.51% (47nd percentile)
	Cβ deviations	0.03%
	Bad backbone	0.06%
	Bad backbone angles	0.19%

* Clash score is the number of serious steric overlaps (>0.4 Å) per 1000 atoms.

**Table 3:** Molprobity Statistics for HPV-U4.

Asymmetric Unit	Six Subunits (L1 Protein)	Cryo-EM Model 3004 Residues at 5.8 Å Resolution after Phenix Global Refinement
Density agreement	Correlation Coefficient	0.82
All-atom contacts	Clash score * (all atoms)	49.58 (4th percentile)
Protein geometry	Poor rotamers	0.25%
	Favored rotamers	98.75%
	Ramachandran outliers	1.33%
	Ramachandran favored	91.09%
	Molprobity Score	2.69% (47nd percentile)
	Cβ deviations	0.13%
	Bad backbone	0.18%
	Bad backbone angles	0.34%

* Clash score is the number of serious steric overlaps (>0.4 Å) per 1000 atoms.

**Table 4:** The conformational change of each L1 protein (see Figure 5A for L1 labels) includes both rotational (degree) and translational (Angstrom) movements.

**HPV-V5**	**Pentavalent Capsomer L1**					**Hexavalent Capsomer L1**				
	#1	#2	#3	#4	#5	#a	#b	#c	#d	#e
Angle (◦)	0.21	0.22	0.22	0.21	0.55	0.20	0.54	0.42	0.48	0.27
Distance (Å)	2.99	2.28	0.99	1.61	2.51	1.57	1.50	2.45	2.57	2.25
**HPV-U4**	**Pentavalent Capsomer L1**					**Hexavalent Capsomer L1**				
	#1	#2	#3	#4	#5	#a	#b	#c	#d	#e
Angle (◦)	0.96	0.96	0.96	0.96	0.97	1.39	1.00	0.64	0.93	1.37
Distance (Å)	1.98	1.98	1.99	2.00	3.45	1.25	1.68	2.36	2.86	1.82

**Table 5:** Residues that comprise the conformational epitopes of V5 and U4. Residues from neighboring L1 protein was designated by X′.

L1 Protein Surface Loop and C-Terminal Arm (C-Ter)			V5 Fab Heavy Chain Outwards	U4 Fab Heavy Chain Count Clockwise
BC	55	PRO	X	
DE	139	ALA	X′	
	142	ASP	X	
	143	ASN	X	
EF	176			X
	179			X
	180			X
	181	ASN	X′	
	182	PRO	X′	
FG	267	VAL	X′	
	278	LYS	X′	
	280	SER	X′	
	282	SER	X′	
	285	ASN	X′	
HI	348	ILE	X	
	357	ASN	X	
	358	THR	X	
C-TER	431	HIS		X′
	437	LYS		X′
